# A dark umbilical nodule in a postmenopausal woman: What is your diagnosis?

**DOI:** 10.51866/tyk.453

**Published:** 2025-07-14

**Authors:** Nur Hidayah Mohd Fauzi, Nik Ahmad Zuky Nik Lah, Siti Masitah Hamzah, W Fadhlina W Adnan, Adibah Ibrahim, Noorul Balqis Che Ibrahim, Engku Ismail Engku-Husna

**Affiliations:** 1 MD, MMed OBGYN, Department of Obstetrics and Gynaecology, School of Medical Sciences, Universiti Sains Malaysia, Kubang Kerian, Kelantan, Malaysia. Email: enhusna@usm.my; 2 MD, MMed OBGYN, Department of Obstetrics and Gynaecology, School of Medical Sciences, Universiti Sains Malaysia, Kubang Kerian, Kelantan, Malaysia.; 3 Department of Obstetrics and Gynaecology, School of Medical Sciences, Universiti Sains Malaysia, Kubang Kerian, Kelantan, Malaysia.; 4 MD, Department of Obstetrics and Gynaecology, School of Medical Sciences, Universiti Sains Malaysia, Kubang Kerian, Kelantan, Malaysia.; 5 MBBS, MMed OBGYN, Department of Obstetrics and Gynaecology, School of Medical Sciences, Universiti Sains Malaysia, Kubang Kerian, Kelantan, Malaysia.; 6 MD, MMed OBGYN, Department of Obstetrics and Gynaecology, School of Medical Sciences, Universiti Sains Malaysia, Kubang Kerian, Kelantan, Malaysia.; 7 MD, MMed Pathology, Department of Pathology, School of Medical Sciences, Universiti Sains Malaysia, Kubang Kerian, Kelantan, Malaysia.

**Keywords:** Umbilical neoplasm, Neoplasm metastasis, Endometrial neoplasms, Primary health care, Diagnostic imaging

## Abstract

Sister Mary Joseph’s nodule is a rare but significant clinical sign that often indicates advanced intraabdominal malignancy. It presents as a palpable umbilical nodule and may be the first indication of an occult primary cancer. In this article, we discuss the case of a 58-year-old postmenopausal woman who presented to a primary care clinic with a darkened umbilical lesion and progressive abdominal symptoms. She wps ultimately diagnosed with metastatic endometrial carcinoma. This uncommon presentation highlights the importance of a comprehensive approach to umbilical lesions in the primary care setting. Recognising this subtle sign in primary care can lead to expedited investigations and timely intervention.

## Case summary

**Figure 1:**
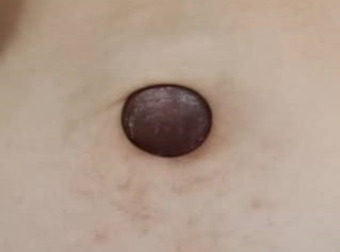
The umbilicus of a postmenopausal woman showing a well-defined, firm, hyperpigmented nodule measuring approximately 4 cm in diameter.

A 58-year-old postmenopausal woman with known type 2 diabetes mellitus and hypertension presented to a primary care clinic with complaints of progressive abdominal distension over 3 months. She also reported unintentional weight loss, poor appetite and general lethargy. Notably, she had experienced postmenopausal bleeding 2 months prior but had not sought medical attention. Two weeks before the visit, she had noted a gradually enlarging, dark-coloured nodule over her umbilicus. It had felt firm and mildly tender. There was no history of trauma, piercing or discharge from the lesion. On examination, the umbilical nodule was approximately 4 cm, dark and fixed (Figure 1). Her abdomen was distended with shifting dullness, but no signs of hernia were present.

Initial blood tests, including a full blood count and renal and liver profile tests, were performed. The patient was referred for urgent imaging. Abdominal ultrasonography revealed moderate ascites and an enlarged uterus with irregular endometrial thickening. Computed tomography (CT) confirmed a large uterine mass with posterior wall perforation and multiple intraabdominal metastases. Fine-needle aspiration of thy umbilical lesion demonstrated metastatic adenocarcinoma of endometrial origin. Histopathology confirmed International Federation of Gynecology and Obstetrics (FIGO) grade 2 endemetrioid carcinoma.

**Questions:**

1. What is your provisional diagnosis?
2. What further history would you elicit and clinical examinations would you conduct?
3. What investigations would you offer?
4. What would your management plan be?
5. Is there an effective way to prevent this condition in older adults?

**Answers**

1. Figure 1 shows an umbilical nodule that is highly suggestive of Sister Mary Joseph’s nodule (SMJN). This lesion was the initial physical sign that led to the diagnosis of advanced endometrial carcinoma. SMJN is a rare occurrence, accounting for approximately 1%-3% of abdominopelvic malignancies spreading to the umbilicus.^1^ It refers to an umbilical nodule that occurs due to the spread of malignant tumours in the pelvic or abdominal cavity. The typical presentation includes an umbilical or paraumbilical nodule with a firm consistency that ranges in size from 0.5 to 15 cm.^2^ Although SMJN is an uncommon sign, it holds significance as a physical finding. Given its nonspecific cutaneous appearance and rarity, SMJN can be easily overlooked or misdiagnosed.
2. In primary care, history-taking should cover gynaecological (e.g. postmenopausal bleeding or hormonal therapy), gastrointestinal, urinary and constitutional symptoms, along with any skin trauma near the umbilicus and a personal or family history of cancer.
  Red-flag symptoms (e.g. loss of appetite or weight) or a history of malignancy should raise suspicion for advanced or recurrent malignancy. Umbilical lesions may be misdiagnosed as hernias, especially if reducible. Skin trauma may suggest a haematoma or keloid. Inflammatory conditions, such as rheumatoid arthritis and inflammatory bowel disease, may suggest pyoderma gangrenosum. In this case, the patient’s postmenopausal bleeding, 3-month history of progressive abdominal distension, weight loss and reduced effort tolerance gave cause for concern about the possibility of intra-abdominal malignancy. She later developed a dark umbilical nodule that grew to 4 cm over 2 weeks and thus prompted further investigation.
3. In primary care, the initial evaluation should include a thorough physical examination, full blood count, renal and liver function tests, tumour marker assessment (e.g. CA-125, if available) and pelvic or abdominal ultrasonography. At the tertiary level, further investigations to identify the primary malignancy should include transvaginal ultrasound; contrast-enhanced CT of the thorax, abdomen and pelvis; fine-needle aspiration cytology of the umbilical lesion; and endometrial biopsy.
  The ovaries are the most common primary site (25.4%), followed by the colorectal (17.9%), pancreatic (6.5%) and gastric regions (5.9%).^3^ Among the extra-abdominal sources, the breasts are the most frequent site (2.9%). Most patients are in their fifth or sixth decade of life, with a mean age of 61±6 years. Umbilical metastases are more common in women (female-to-male sex ratio of 1.7:1), largely due to the high prevalence of ovarian cancer.^3^ In 14%-33% of cases, the detection of umbilical metastases leads to the diagnosis of previously concealed neoplasms.^4^ In this case, advanced endometrial cancer was suspected based on high-risk factors (i.e. postmenopausal bleeding, diabetes mellitus and hypertension) and suspicious ultrasonography findings (i.e. uterus with thickened endometrium and ascites). Histological examination confirmed endometrial carcinoma of the endometrioid type, FIGO grade 2.
4. In primary care, recognising red flags, providing supportive care (e.g. analgesia) and conducting urgent referrals lead to timely diagnoses and improved outcomes. At the tertiary level, management involves a multidisciplinary team, which includes oncologists, surgeons and palliative care specialists. Treatment may include surgery, chemotherapy, radiotherapy or palliative care depending on the disease extent. SMJN commonly indicates widespread metastasis, and palliative treatment is typically administered. Survival after the diagnosis of an umbilical metastasis is abysmal, with a median survival of less than 8 months.^5^ Different treatment approaches have been reported, including wide excision with an extensive search for the primary lesion, radiotherapy and surgery with adjuvant therapy.^4,6-8^ Survival rates have been found to be higher for patients with primary ovarian (18 months) and endometrial cancers (9 months).^3^ A reported survival of 21 months after surgery supports the application of an aggressive surgical approach in otherwise fit patients.^9^ In patients who undergo aggressive treatment involving a combination of surgery and adjuvant therapy, a survival of 17.6 months has been reported, which surpasses the survival rates of surgery alone (7.4 months), adjuvant therapy alone (10.3 months) and no treatment (2.3 months).^4^
5. There is no known effective way to prevent SMJN, as it is a sign of advanced malignancy. Early identification in primary care can lead to earlier referral and diagnosis. Reducing the risk of endometrial cancer involves weight reduction and the optimisation of diabetes mellitus and hypertension management. Educating patients to seek early medical attention for abnormal symptoms (e.g. postmenopausal bleeding and abdominal distension) can lead to earlier diagnosis and improved outcomes.
